# Genotoxicity of Natural Water during the Mass Development of Cyanobacteria Evaluated by the Allium Test Method: A Model Experiment with Microcosms

**DOI:** 10.3390/toxins14050359

**Published:** 2022-05-21

**Authors:** Dmitry S. Pesnya, Svetlana A. Kurbatova, Andrey N. Sharov, Ekaterina N. Chernova, Igor Y. Yershov, Galina V. Shurganova, Ekaterina L. Vodeneeva

**Affiliations:** 1 Laboratory of Water Ecosystems, Department of Ecology, Institute of Biology and Biomedicine, Lobachevsky State University, 603022 Nizhny Novgorod, Russia; kurb@ibiw.ru (S.A.K.); ershov@ibiw.ru (I.Y.Y.); galina.nngu@mail.ru (G.V.S.); vodeneeva@mail.ru (E.L.V.); 2 Laboratory of Experimental Ecology, Papanin Institute for Biology of Inland Waters, Russian Academy of Sciences, 152742 Borok, Russia; 3 Laboratory of Algology, Papanin Institute for Biology of Inland Waters, Russian Academy of Sciences, 152742 Borok, Russia; sharov@ibiw.ru; 4 Laboratory of Bio-Electronic Methods of Geo-Ecological Monitoring, St. Petersburg Federal Research Center of the Russian Academy of Sciences (SPC RAS), Scientific Research Centre for Ecological Safety of the Russian Academy of Sciences, 197110 St. Petersburg, Russia; 5 Laboratory of Eco-Chemical Studies, St. Petersburg Federal Research Center of the Russian Academy of Sciences (SPC RAS), Scientific Research Centre for Ecological Safety of the Russian Academy of Sciences, 197110 St. Petersburg, Russia; s3561389@yandex.ru

**Keywords:** genotoxicity, cyanobacteria, harmful cyanobacterial blooms, cyanotoxins, microcystins, microcosms, Allium test

## Abstract

Cyanobacteria, which develop abundantly in aquatic ecosystems, can be harmful to humans and animals not only by releasing toxins that cause poisoning but also by provoking cytogenetic effects. The influence of the mass development of cyanobacteria on the genotoxic properties of natural water has been studied in model ecosystems (microcosms) with different compositions of biotic components (zooplankton, amphipods and fish). The validated plant test system “Allium test” was used in this study. Genotoxic effects were detected at microcystin concentrations below those established by the World Health Organization (WHO) for drinking water. In all experimental treatments, cells with disorders such as polyploidy and mitotic abnormalities associated with damage to the mitotic spindle, including c-mitosis, as well as lagging chromosomes were found. Genotoxic effects were associated with the abundance of cyanobacteria, which, in turn, depended on the composition of aquatic organisms in the experimental ecosystem. Fish, to a greater extent than other aquatic animals, maintain an abundance of cyanobacteria. After one month, in microcosms with fish, mitotic abnormalities and polyploidy continued to be detected, whereas in other treatments, there were no statistically significant genotoxic effects. In microcosms with amphipods, the number and biomass of cyanobacteria decreased to the greatest extent, and only one parameter of genotoxic activity (frequency of polyploidy) significantly differed from the control.

## 1. Introduction

The mass development of cyanobacteria in water bodies is a global problem that is increasing with the eutrophication of aquatic ecosystems and climate warming [1]. Many species of cyanobacteria are considered potentially toxic. There are cases all over the world where the “blooming” water of reservoirs caused by the mass development of cyanobacteria has led to the acute intoxication of people and animals [2,3,4]. The most common cyanotoxins are neurotoxic or hepatotoxic compounds [5]. Microcystins (MCs) are the most common hepatotoxic substances produced by cyanobacteria in freshwater ecosystems [6]. These are persistent cyclic peptide hepatotoxins. MCs accumulate in different compartments of aquatic environments, including fish, mussels and sediment. These compounds are highly toxic, and different exposure routes are possible [7]. To date, more than 279 MCs variants have been described [8].

According to a recent database, more than 2000 secondary metabolites are currently reported, including many cyanopeptides. Among them, many may pose a risk to human health, and other metabolites are considered as potential pharmacological substances for the treatment of some major diseases (infections, cardiovascular diseases and cancer). Among the known secondary metabolites are cyanotoxins such as microcystins, saxitaxins, cylindrospermopsins, nodularins and anatoxin-a. These cyanotoxins can cause serious harm to various human organs: the liver, nervous system and brain, skin and gastrointestinal tract. However, little is known about the potential genotoxicity and human risks posed by exposure to less well-studied secondary metabolites [9]. In addition to toxic effects on various organs and systems of animals and humans, secondary metabolites of cyanobacteria cause cytogenetic effects [10,11,12]. This aspect of the action of cyanotoxins has been studied to a lesser extent than toxicity in the general sense. MCs cause the inhibition of protein phosphatases [13,14], which leads to the hyperphosphorylation of proteins and ultimately to various cytogenetic effects. Genotoxic effects are explained by the formation of reactive oxygen species (ROS). It has been established that MCs can lead to the formation of malignant tumors [15]. In studies on mammalian and human cells, as well as on rodents in vivo, it has been shown that MCs induce the appearance of ROS and cause DNA damage and the formation of micronuclear mutations in cells [10]. Cyanotoxins can manifest themselves as genotoxic or mutagenic agents. However, this depends on different parameters: exposure concentrations, time of exposure, test systems, pure material or in complex mixtures and other conditions. The mutagenic and/or genotoxic effects of cyanotoxins such as MCs, cylindrospermopsins, nodularins and saxitoxins have been shown in several studies [16,17,18,19].

Environmental pollution by mutagens is very dangerous. Such pollution can cause hereditary and oncological diseases, congenital malformations, decreased immunity, premature aging and autoaggressive diseases [19,20,21,22]. Due to its biological significance, genotoxicological studies should be the main focus when biomonitoring water reservoirs in which there is a massive development of cyanobacteria. In the scientific literature, various studies have been presented in which the mutagenic and genotoxic effects of different cyanotoxins have been evaluated both individually and simultaneously using various test models: both prokaryotic and eukaryotic. However, additional research is needed for the better understanding of cyanotoxin behaviors in different conditions and concentrations, as well as co-interactions in natural mixtures and various ecosystems. In addition, the underlying mechanisms of genotoxicity are not entirely clear [16,17,18,19,23,24,25].

To understand the real effects produced by secondary metabolites of cyanobacteria on the genetic apparatus and the process of cell division of living organisms, it is not sufficient to determine the dependence of “the dose of the toxicant–cytogenetic effect” in the laboratory. Under natural conditions, cyanobacteria enter various relationships with other biotic components of ecosystems. As a result, cyanobacteria metabolites, including cyanotoxins, undergo transformation. Depending on the conditions, these substances can degrade or accumulate at different rates and are transmitted along trophic chains [26,27]. Some studies show that cyanotoxins in natural water can be more toxic than purified material [11]. Thus, studies of cytogenotoxic effects should also be carried out directly in aquatic ecosystems.

The Allium test is an accessible, convenient, fast, sensitive and easily reproducible cytogenetic bioassay for determining genotoxic effects. It registers different cytogenetic and genotoxic parameters such as the mitotic index, phase indices, chromosomal aberration, mitotic abnormalities and micronuclei. The use of the Allium test as a bioindicator has been validated and standardized by the International Program on Plant Bioassays (IPPB) of the United Nations Environment Program (UNEP) and Geno-Tox of the U.S. Environmental Protection Agency (USEPA) [28]. The data obtained from the Allium test are recognized as reliable and effective to detect environmental genotoxicity (in the water, air and soil) by the USEPA and the World Health Organization (WHO). It has been shown that the Allium test is sensitive and reliable to detect the genotoxic and mutagenic potential of any natural/chemical/synthetic compounds [29]. The Allium test is recommended as standard in environmental monitoring and especially for aquatic ecosystem assessment [12,30,31]. When standardizing the method, it is reported that the data obtained in the Allium test can be used to assess genotoxicity not only for plants but for eukaryotes in general, including humans [30]. This is because of the function of storing and transmitting genetic information; the structure of the genetic apparatus is conservative in all eukaryotes, whether plant or animal cells. Allium test data have good correlation with other test systems: algae, fish, bacteria (Ames test), as well as tests on animal cell cultures and human lymphocytes. Thus, the results obtained in the Allium test are sufficient to perform a rapid and reliable genotoxic assessment of various factors (including water samples) in ecotoxicological studies [31]. In addition, the Allium test is recommended for assessment of the cyanotoxin’s genotoxic effects (including MCs) [10,11,12].

The aims of the present study were: (i) to identify the type of cytogenetic and genotoxic effects caused by cyanobacteria blooming in aquatic ecosystems, and (ii) to determine whether cytogenetic effects depend on the complexity of the organization of ecosystems and the composition of their biotic components. This study was carried out using experimental ecosystems (microcosms). The microcosm method enables the researcher to create a physical model of an ecosystem with specified controlled environmental parameters and the necessary composition of biota. In the experiment, microcosms of three levels of organization were created: only with zooplankton (“ZP”), zooplankton and bottom crustaceans (amphipods) (“Amph”) and zooplankton and fish (“F”).

## 2. Results

### 2.1. Number and Biomass of Cyanobacteria

**Figure 1:**
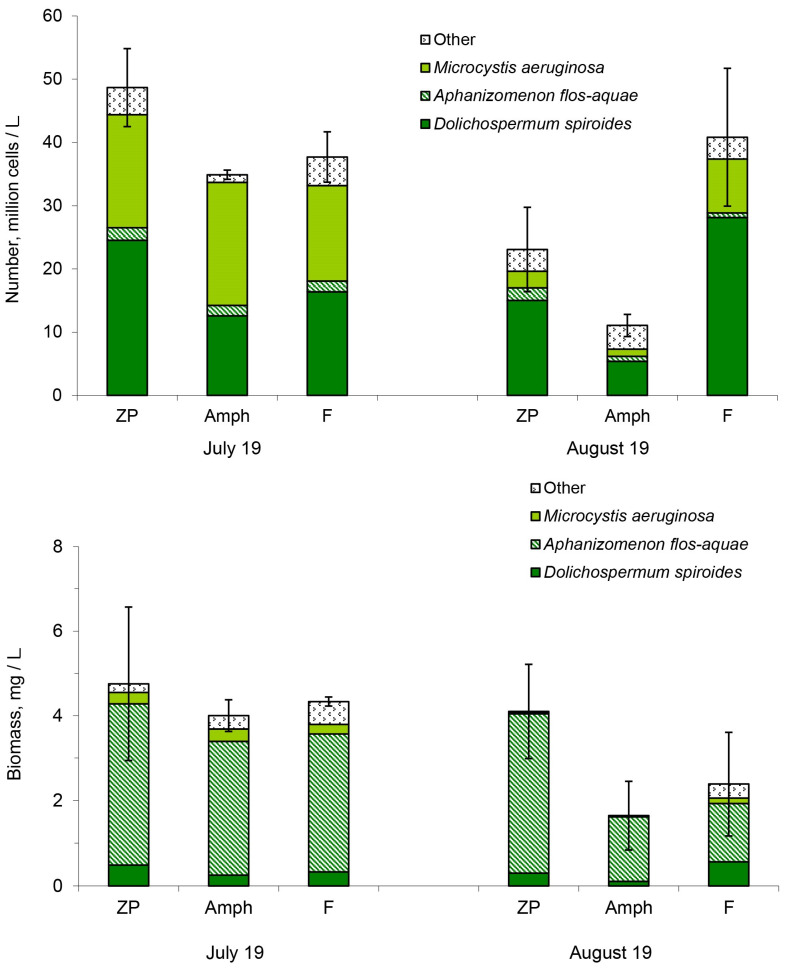
Number and biomass of cyanobacteria in the different treatments of the experiment: ZP, microcosms with only zooplankton; Amph, zooplankton and amphipods; and F, zooplankton and fish. Confidence intervals (95%) for the total number of cyanobacteria are given.

In all microcosms, cyanobacteria account for 98% of the abundance and more than 80% of the biomass of all phytoplankton. In total, 11 species of cyanobacteria have been observed in microcosms. *Dolichospermum spiroides*, *Microcystis aeruginosa* and *Aphanizomenon flos-aquae* were the dominant species. At the beginning of the experiment (19 July 2021), *D. spiroides* and *M. aeruginosa* prevailed in numbers in approximately equal proportions (Figure 1). One month later (19 August 2021), the number of cyanobacteria decreased in the treatments “ZP” and “Amph” and changed insignificantly in the treatment “F”. *D. spiroides* dominated in numbers in all treatments of the experiment in August (Figure 1). The biomass of cyanobacteria became smaller in the treatments “Amph” and “F” at the end of the experiment. *A. flos-aquae* dominated in terms of biomass among all cyanobacteria throughout the experiment (Figure 1).

### 2.2. Microcystin Concentrations

**Figure 2:**
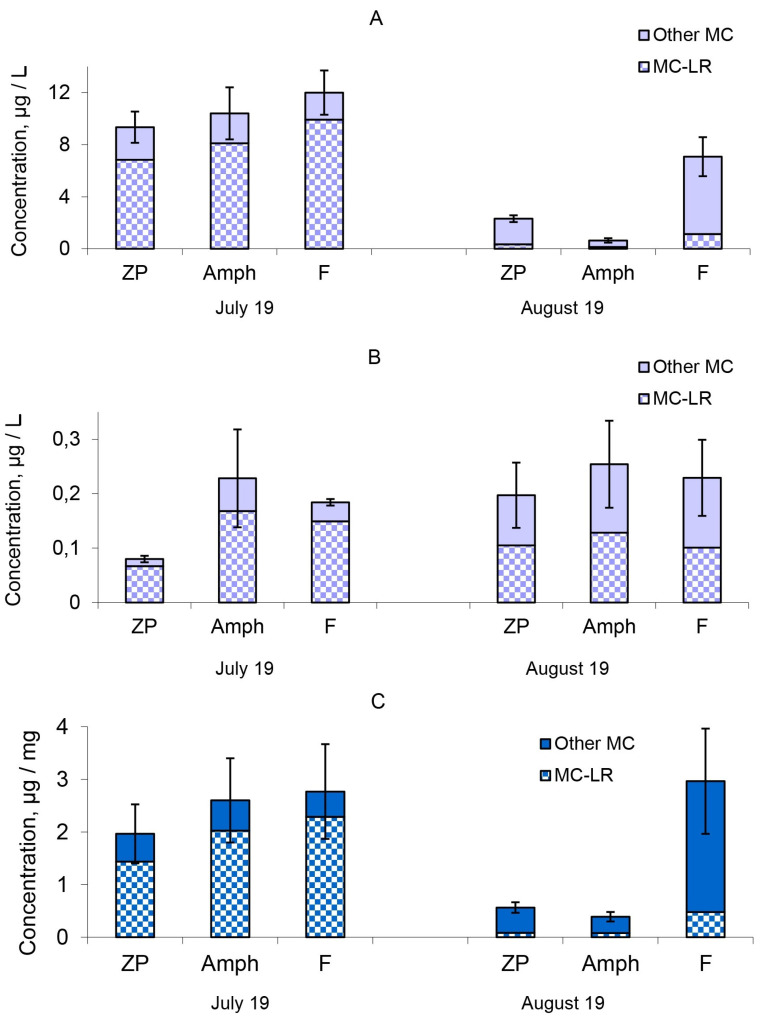
Microcystin concentration: (**A**), in phytoplankton biomass; (**B**), in water; (**C**), MC concentration in one unit (mg) of cyanobacteria biomass. “Other MCs” denote to the sum of other 7 detected MC congeners: [D-Asp^3^]MC-LR, MC-LR, [D-Glu-OCH_3_^6^]MC-LR, [D-Asp^3^]MC-RR, MC-LW, [Dha^7^]MC-YR, MC-YR and MC-RR. Designations of treatments are as in Figure 1. Confidence intervals (95%) for the total concentration of microcystin are given.

The MC concentrations in phytoplankton biomass were 9.34–12.00 μg/L in the different treatments at the beginning of the experiment (Figure 2A). The extracellular MC concentrations in water (Figure 2B) were significantly lower (0.08–0.228 μg/L) than in the phytoplankton biomass (Tables S1 and S2). There were eight structural MCs variants identified: [D-Asp^3^]MC-LR, MC-LR, [D-Glu-OCH_3_^6^]MC-LR, [D-Asp^3^]MC-RR, MC-LW, [Dha^7^]MC-YR, MC-YR and MC-RR (Table S2). The most toxic forms of MCs, MC-LR, prevailed and accounted for 73–84% of the total MC amount (Figure 2). The intracellular MC concentration decreased by one-half in microcosms with fish (“F”) by the end of the experiment (Figure 2A). The intracellular MC concentrations of the “ZP” and “Amph” microcosms decreased to the greatest extent by the end of the experiment (Figure 2A). The lowest MC concentrations were found in microcosms with benthic crustaceans (“Amph”), where the lowest numbers and biomass of cyanobacteria were also registered. The MC concentration contained in 1 mg of cyanobacterial biomass did not decrease after one month of observations in the treatment “F”, but significantly decreased in the other two treatments “ZP” and “Amph” (Figure 2C). In the model ecosystems with fish, at the end of the experiment, the same MC concentrations as at the beginning were focused in a smaller biomass of cyanobacteria. 

### 2.3. Mitotic and Phase Indexes, and Genotoxic Effects

**Figure 3:**
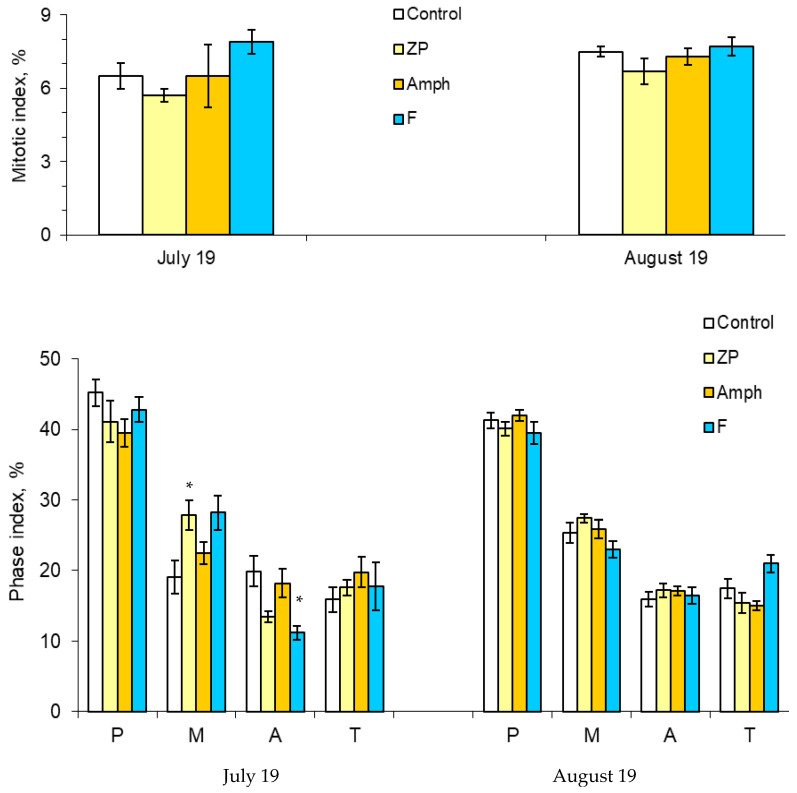
Mitotic and phase indices: P, prophase; M, metaphase; A, anaphase; and T, telophase. Designations of treatments are as in Figure 1. Asterisks (*) mark significant differences from the control (Mann–Whitney test, *p* < 0.05).

**Figure 4:**
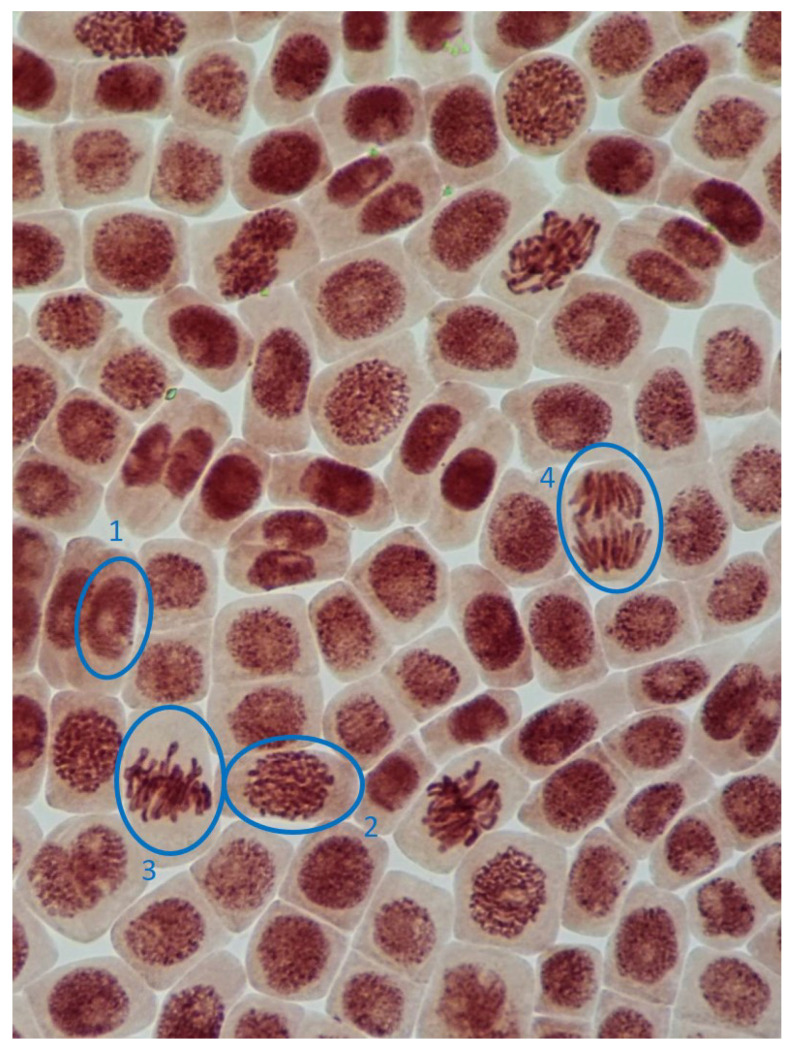
Microphotography of cells in the root tips of *Allium cepa* L. with different phases of mitosis and interphase cells (1, interphase; 2, prophase; 3, metaphase; 4, anaphase).

**Figure 5:**
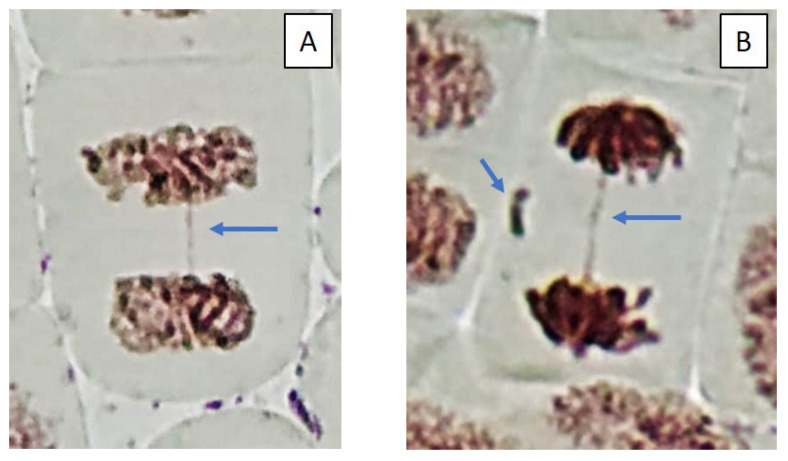

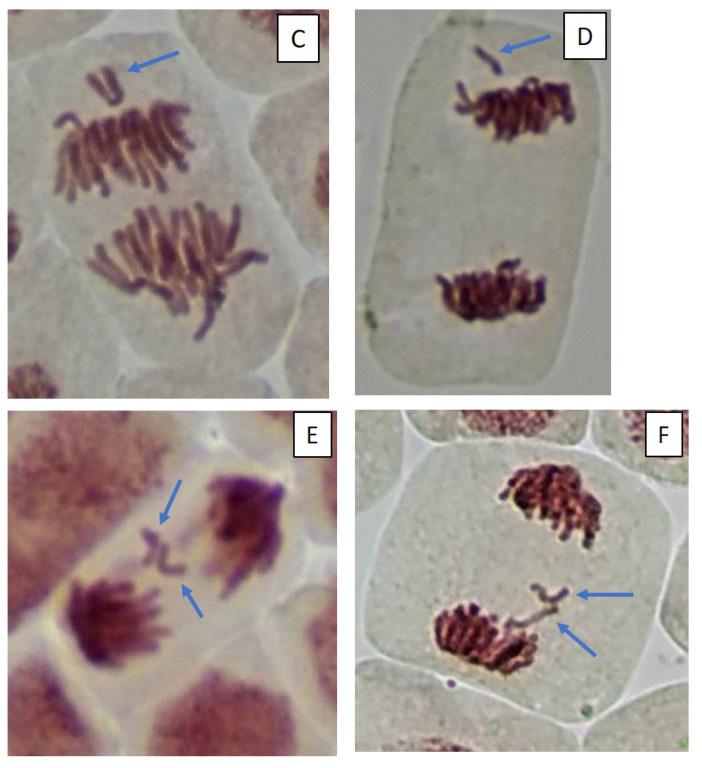

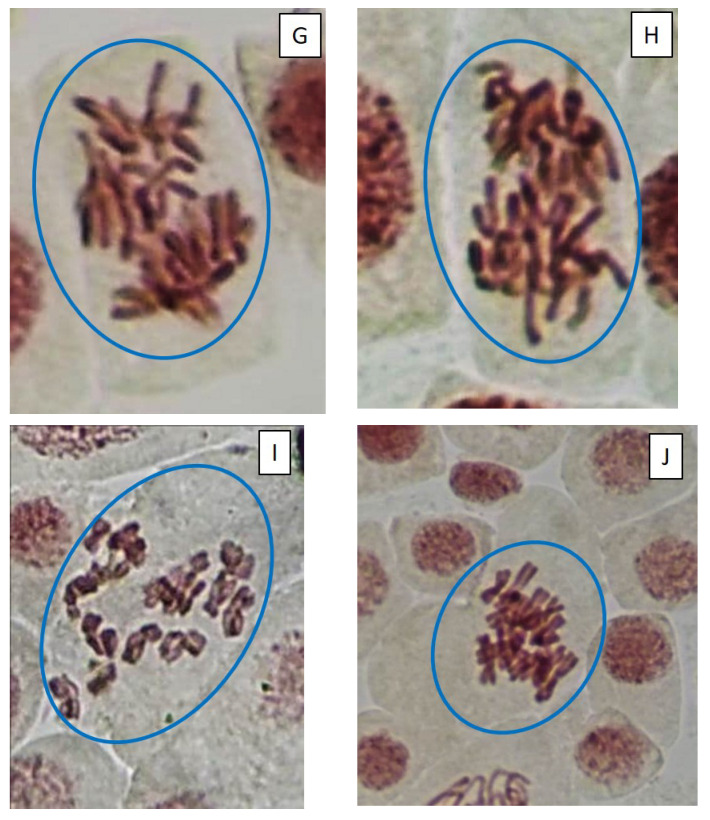

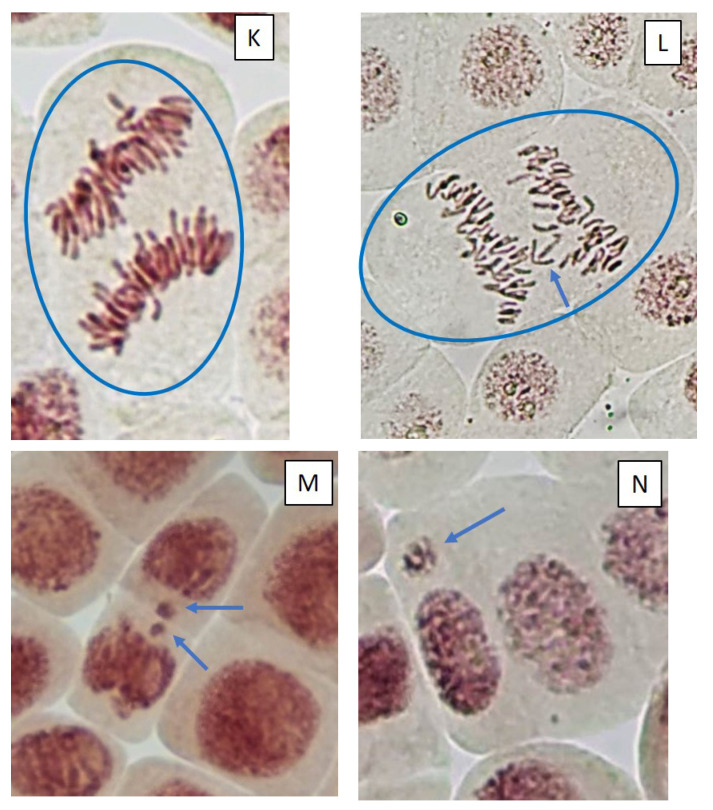
Microphotography of cells in the root tips of *Allium cepa* L. exposed to the water from pools with model microcosms. Location of chromosome aberrations in the cells indicated by arrows. Mitotic abnormalities and polyploidy indicated by circles. Types of abnormalities: (**A**) chromosome bridge; (**B**) chromosome bridge and single fragment; (**C**) vagrant chromosome; (**D**) fragment; (**E**) two lagging chromosomes; (**F**) lagging chromosome and fragment; (**G**) mitotic spindle disturbances; (**H**) mitotic spindle disturbances; (**I**) C-mitosis; (**J**) C-mitosis; (**K**) polyploidy; (**L**) polyploidy with lagging chromosomes; (**M**) small micronuclei (formation from fragments); (**N**) large single micronuclei (formed from lagging chromosome).

The mass development of cyanobacteria in the model ecosystems did not significantly change the number of dividing cells (Figure 3). However, some changes have been noticed in indicators such as the phase indices (Figure 3 and Figure 4). At the beginning of the experiment (July), in the treatment “ZP”, the proportion of cells in the metaphase significantly increased, and in the treatment “F”, the proportion of cells in the anaphase decreased (Figure 3) (Table S3). The situation when the metaphase index is increased may be associated with the action of cyanobacterial toxins on the mitotic spindle. In this case, chromosomal segregation is disrupted, which may result in the appearance of aneugenic events such as lagging chromosomes and polyploidy (Figure 5).

Cells with mitotic abnormalities, chromosomal abnormalities and micronuclei were registered in all the treatments of the experiment. Increasing trends (not statistically significant) in the frequency of micronuclei and chromosomal aberrations were found. Observed micronuclei may be associated with both clastogenic and aneugenic events. The appearance of chromosomal aberrations such as bridges and fragments was registered. There were mitoses with lagging and vagrant chromosomes and mitotic disorders associated with damage to the division spindle. The appearance of genomic mutations, polyploid cells and c-mitoses were registered.

**Figure 6:**
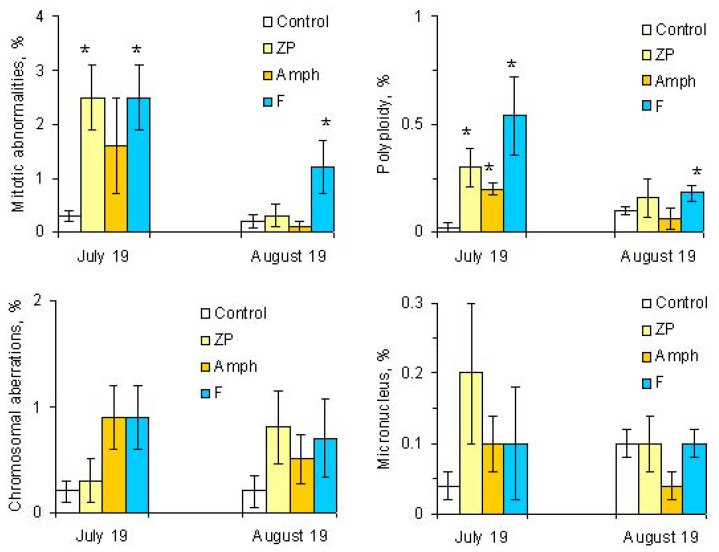
Genotoxic effects in microcosms. Designations of treatments are as in Figure 1. Asterisks (*) mark significant differences from the control (Mann–Whitney test, *p* < 0.05).

Frequencies of mitotic abnormalities were significantly higher than control levels in July in the treatments “ZP” and “F” and after one month, only in the treatment “F”. The most frequent mitosis anomalies were associated with damage to the division spindle. Lagging and vagrant chromosomes and c-mitosis were often observed. In all microcosms with cyanobacteria, cells with polyploidy were recorded to varying degrees (Figure 5 and Figure 6). Clastogenic events (chromosomal aberrations) did not significantly differ from the control. 

After one month, genotoxic effects were not statistically significant in most microcosms. The exceptions were experimental ecosystems with fish, where mitotic abnormalities and polyploidy were still detected more often than in the control (Figure 6).

## 3. Discussion

The concentrations of MCs (0.08–0.228 μg/L) in water in our experiment were similar to levels as a result of the development of cyanobacteria in the natural phytoplankton community under natural conditions. Low concentrations of MCs in the range of 0.1–1 μg/L are often recorded in natural water bodies in Europe during the mass development of cyanobacteria [32,33]. Under the conditions of this experiment, concentrations of cyanobacterial cells were 35–49 million cells/L (Figure 1). According to the WHO’s cyanobacteria cell abundance levels, this can be categorized as a moderate (20,000,000 ≤ cells/L ≤ 100,000,000) exposure health risk [34]. In the water of the experimental ecosystems, genotoxic effects were observed at MC concentrations below those established by the WHO for drinking water (<1 μg/L) [35]. This indicates the danger of cytogenetic abnormalities even at low concentrations of cyanotoxins in water. It is assumed that genotoxic effects are not caused only by known cyanotoxins but also by other secondary metabolites of cyanobacteria because cytogenetic anomalies have been observed as a result of the development of cyanobacteria strains that do not produce toxins [10]. It has been shown that MC-LR in natural water during the mass development of cyanobacteria is more toxic than the pure MC-LR substance, which may be explained by synergistic interactions with other compounds [11].

In the present study, it was found that even naturally occurring low concentrations (less than 1 μg/L) of MCs were associated with the disruption of cell division processes, causing mitotic arrest in the cell cycle metaphase. Such violations can lead to the development of tetraploidization, followed by further polyploidization [36]. In some studies, the polyploidization of MC-treated cells has been registered, which may be related to the disruption of the mitotic spindle [37]. In the present experiment, mitotic abnormalities and polyploidy were observed, which may also be associated with mitotic spindle inhibition. Polyploidy might be considered a potential initiator of tumor growth and a mechanism of resistance to different toxic substances and stressful conditions [36,38]. Due to mitotic anomalies such as lagging and vagrant chromosomes, c-mitosis indicates a risk of chromosomal instability and aneuploidy [31]. It has been shown that aneuploidy is one of the hallmarks of cancer [38].

Our data have some similarities to other laboratory and field studies performed using the Allium test. Methanolic extracts from the NPLJ-4 strain of *M. aeruginosa* with very high concentrations of MCs (65.8–6580 µg/L) induced both clastogenic and aneugenic effects (mitotic abnormalities, chromosome aberrations and micronuclei) [10]. In another study, genotoxic effects were observed after exposure to MS-LR concentrations 1–2 μg/L (laboratory pure substance) and 6.23–6.88 μg/L (in natural water samples collected in the Salto Grande reservoir during a cyanobacterial bloom). The authors only highlighted clastogenic effect for the treatments, but aneugenic effects are also clear from their Allium test data [11]. However, in our study, we did not observe clastogenic effects at low MC concentrations (0.08–0.228 µg/L) but only mitotic abnormalities, lagging chromosomes and c-mitosis, associated with aneugenic activity.

Thus, the obtained data indicate that even MC concentrations below WHO levels in natural waters may pose a threat to the health of aquatic organisms. Additionally, this may pose a risk to human health. However, more research needs to be performed using additional in vitro and in vivo tests to reach a definitive conclusion.

In ecosystems with aquatic animals, the content of cyanotoxins in water can change rapidly. On the one hand, aquatic animals are able to reduce the number of cyanobacteria by feeding on them [39,40]. On the other hand, aquatic organisms potentially contribute to an increase in the concentration of cyanotoxins in water. The intracellular concentration of MCs during the study was significantly higher than in water. Cyanotoxins are released into water during cell lysis. The destruction of cyanobacterial cells occurs with the active participation of aquatic animals. For example, zooplankton can consume cyanobacteria for food [41,42]; as a result, cyanobacterial cells are destroyed, and cyanotoxins enter the water. In addition, algophages promote cyanotoxin production through an increase in the abundance of cyanobacteria. Aquatic organisms eat algae more suitable for nutrition and thereby eliminate competitors for cyanobacteria [43]. In addition, hydrobionts of all ecological groups (plankton, benthos and fish) secrete phosphorus in a form easily accessible to primary producers [44], which accelerates the turnover of nutrients in the ecosystem and promotes their rapid use by cyanobacteria and algae. It is possible that in this experiment, allelopathic interactions also directly or indirectly affected the concentration of MC, as well as the dynamics of changes in the biomass and abundance of cyanobacteria species [45].

The acute toxicity of cyanobacteria metabolites has rarely been observed in relation to aquatic animals. The coexistence of aquatic animals and cyanobacteria over a very long period of time has led to the development of mutual adaptations. For example, zooplankton develops both physiological or behavioral reactions and genetic tolerance in response to the adverse effects of cyanobacteria [46]. However, under certain conditions, oxidative damage and the inhibition of protein phosphatases are also exhibited in planktonic crustaceans (*Daphnia*) [14].

The degree of manifestation of mitotic abnormalities and polyploidy was dependent on the abundance of cyanobacteria in the experimental ecosystems. To a greater extent, genotoxic effects were recorded at the beginning of the experiment, along with a significant number and biomass of dominant cyanobacteria species. One month later, when the number of cyanobacteria (especially *Microcystis aeruginosa*) decreased in the “ZP” and “Amph” treatments, the genotoxic effects were weak and did not differ from the control. At the same time, in the treatment with fish (“F”), where a higher number of *M. aeruginosa* remained, the frequency of polyploidy and mitotic anomalies were higher than the control. The most probable reason for stimulating the development of cyanobacteria by fish is the excretion of a significant amount of labile phosphorus. It is known that phosphorus excretion is associated with the mass of aquatic animals; fish, having a large total biomass in ecosystems in comparison with aquatic animals of other ecological groups, often secrete a larger amount of phosphorus [47]. In addition, it has been shown that the passage of cyanobacteria through the digestive tract of animals can further stimulate their development. A comparison between fish, mollusks and zooplankton showed that the passage of cyanobacteria through the intestines of fish provokes "blooming" to the greatest extent [48].

One month later, in microcosms with the benthic crustaceans amphipods (“Amph”), the decrease in the abundance of cyanobacteria was more significant than in other treatments. At the same time, the genotoxic effects were weaker. Although statistical analysis did not show significant differences from other treatments, it seems that we can discuss a tendency to decrease genotoxicity in the presence of amphipods. The importance of amphipods in regulating the abundance of cyanobacteria and in changing the concentration of cyanotoxins in natural water requires dedicated research in the future. Perhaps amphipods are more efficient consumers of cyanobacteria. They “extracted” cyanobacteria, as well as the toxins contained in them, from the water column and transferred them to bottom sediments. It is known that significant fractions (35–73%) of dissolved MCs are absorbed in sediments shortly after being released from cyanobacterial cells [49]. Amphipods probably accelerate this process.

## 4. Conclusions

Genotoxic effects, as a consequence of the mass development of cyanobacteria (35–49 million cells/L), can be detected even at low concentrations (0.08–0.228 µg/L) of MCs in natural water, which is below WHO guideline values for drinking water (1 µg/L). Frequencies of chromosomal aberrations and micronuclei were not statistically significant in all treatments of the experiment; thus, no clastogenic activity was observed at 0.08–0.228 μg/L MC concentrations. The most common cytogenetic abnormalities in all treatments of microcosms were mitotic disturbances, lagging/vagrant chromosomes, c-mitosis and polyploidy, which is associated with aneugenic activity. Changes in mitotic indices indicate mitotic arrest in the metaphase of the cell cycle. Therefore, the mechanism of genotoxic effects may be associated with chromosome mis-segregation and damage to the mitotic spindle. Thus, the obtained data indicate that natural water with concentrations of MCs 0.08–0.228 µg/L may exert aneugenic activity and lead to chromosomal instability.

The results of the present study indicate that cytogenetic effects depend on the complexity of an ecosystem’s organization and the composition of its biotic components. Aquatic animals of different ecological groups influenced the genotoxic effects through changes in the abundance of cyanobacteria.

In microcosms with fish, these aquatic animals contributed to the maintenance of a larger number of cyanobacteria and higher MC concentrations in total phytoplankton biomass and in one unit of cyanobacteria biomass at the beginning of the experiment (19 July 2021), as well as after one month of the experiment (19 August 2021), which resulted in the longer manifestation of genotoxic effects. Only in microcosms with fish were significant increases in the frequencies of mitotic abnormalities and polyploidy after one month of the experiment. Thus, fish may contribute to the accumulation of cyanobacteria and cyanotoxins in ecosystems.

In microcosms with amphipods, the number and biomass of cyanobacteria decreased notably more than in other treatments, and only one parameter of genotoxic activity was significantly higher than in the control at the beginning of the experiment. No genotoxic effects were registered after one month of the experiment. Thus, amphipods may contribute to the elimination of cyanobacteria and cyanotoxins from the ecosystem.

Determination of the influence of aquatic animals of various trophic and ecological groups on the strength and duration of genotoxic effects as a result of the mass development of cyanobacteria requires additional research. This will help to more accurately determine in which ecosystems there is faster purification from genotoxicants.

## 5. Materials and Methods

### 5.1. Microcosms Organization

Model ecosystems (microcosms) with different compositions of their biotic components were formed in plastic fish-breeding containers (1 × 1 × 0.5 m) (Figure S1). Water from the Sunoga River (58.0404° N, 38.2412° E) (300 L) was poured into the microcosms. Algae and microorganisms, as well as some zooplankton, entered the microcosms together with natural water. The abundance of zooplankton was increased by the additional introduction of plankton from artificial ponds filled with the same river water. The initial number of zooplankton was 562 ± 68 ind./L. The microcosm communities were dominated by a large filter-feeder, *Daphnia longispina*. The cladoceran *D. longispina* accounted for approximately 37% of the total abundance. Cyanobacteria concentrates taken from a pool with cyanobacterial blooming were introduced into each microcosm (Figure S2). The microcosms were placed outdoors in natural light in a concrete pool filled with water to prevent sudden temperature spikes during the day (Figure S1). The experiment included three treatments: “ZP”, microcosms with zooplankton only; “Amph”, microcosms which were additionally populated with 150 specimens of the bottom crustacean amphipod *Hyalella azteca* (individual weight 2.95 ± 0.4 mg); and “F”, microcosms with *Oryzias latipes* fish (15 specimens per microcosm, individual weight 461 ± 38 mg) [50]. Each treatment was replicated three times. The experiment lasted for one month, from 19 July to 19 August 2021.

### 5.2. Phytoplankton

Phytoplankton was taken in 0.5 L plastic bottles for analysis of the composition of algae and plankton bacteria and conserved with a few drops of Lugol solution with the addition of formalin, ice acetic and chromic acids. Samples were left for sedimentation for 10 days and were then siphoned off to reduce them to 10 mL.

Species identification and individual cell counting were carried out in a Nageotte chamber with a volume of 0.02 mL using a Bioptic B-200 optical microscope (Biomed, St. Petersburg, Russia) at 420× and 600× magnifications. The biomasses of microalgae and cyanobacteria were calculated from the sum of the biovolume of all cells in the subsample, assuming that 10^9^ μm^3^ corresponded to 1 mg of phytoplankton wet biomass [50]. Biovolume is expressed in μm^3^ for each counted species, and reflects individual cell volumes; for colony-forming taxa such as diatoms and cyanobacteria, the biovolume is for individual cells, not the size of colonies [51]. The dominant species exceeded 5% of the total abundance and 10% of the total biomass.

### 5.3. Cyanotoxin Analysis

The concentration of MCs was recorded both in phytoplankton biomass (intracellular fraction) and in the water (extracellular fraction). For this purpose, water samples from microcosms were filtered through cellulose acetate membrane filters with pore size 1.2 μm (trade mark “Vladisart”, Vladimir, Russia). The analysis was conducted without animals. Large animals and random debris were removed from the filter. Small zooplankton species could remain in the filter in small numbers but could not introduce a serious error in the measurement. Phytoplankton algae were also not removed from the sample. Then, MCs were separately analyzed in the biomass of phytoplankton deposited on the filters and in the filtrate.

All chemicals used for analytical procedures were of analytical grade. Acetonitrile (HPLC-grade) and methanol (Li Chrosolvhypergrade for LC-MS) were purchased from Merck (Darmstadt, Germany); formic acid (98–100%) was obtained from FlukaChemika (Buchs, Switzerland). High-quality water (18.2 MΩ cm^−1^) was produced with a Millipore Direct-Q water purification system (Bedford, MA, USA). The MC-LR, MC-RR and MC-YR standards were purchased from Sigma Aldrich; MC-LY, MC-LA, MC-LW, MC-LF, [D-Asp^3^]MC-LR and [D-Asp^3^]MC-RR were from Enzo Life sciences, Inc., New York, NY, USA.

The high-performance liquid chromatography–high-resolution mass spectrometry (HPLC–HRMS) method was used to check the presence of cyanotoxins. The sample preparation procedures were performed according to [52]. The samples were filtered using Whatman GF/C filters. The filters with biomass were immediately frozen at −20 °C until extraction. Cyanotoxins from water samples extracted using solid phase extraction Oasis HLB cartridges (60 mg, Waters, Milford, MA, USA). The toxins were eluted from the cartridges with 10 mL methanol. The collected extracts were dried using a rotary evaporator and stored at −20 °C until further analysis. The dried extracts were reconstituted in 1 mL of 80% aqueous methanol and centrifuged (CM-50 centrifuge, ELMI, Riga, Latvia) at 14,000 rpm for 10 min prior to analysis. 

Cyanotoxins from the collected biomass samples on the filters were extracted by treatment with two portions of 75% aqueous methanol under the action of ultrasound after a threefold cycle of freezing–thawing moistened with distilled water filters. The volume of each extract was 2 mL. The extracts were centrifuged (CM-50 centrifuge, ELMI, Riga, Latvia) at 14,000 rpm for 10 min, and aliquots (0.2 mL) were taken for analysis. 

Analyses of extracts were performed using the LC-20 Prominence HPLC system (Shimadzu, Japan) coupled with an LTQ Orbitrap XL Hybrid Ion Trap-Orbitrap Mass Spectrometer (Thermo Fisher Scientific, San Jose, CA, USA), according to [52]. Separation of the toxins was performed on a ThermoHypersil Gold RP C18 column (100 mm × 3 mm, 3 μm) with a Hypersil Gold drop-in guard column (Thermo Fisher Scientific) by gradient elution (0.2 mL min^−1^) with a mixture of water and acetonitrile, both containing 0.05% formic acid.

Mass spectrometric analysis was carried out under conditions of electrospray ionization in positive ion detection mode. The identification of target compounds was based on the accurate mass measurement of [M + H]^+^ or [M + 2H]^2+^ ions (resolution of 30,000, accuracy within 5 ppm), the collected fragmentation spectrum of the ions and the retention times. 

The internal standard isotope-labeled microcystin LR (deuterated, 50 ng/mL) was used for quantitative determination. Limits of detection for different microcystin congeners (2–6 ng/L) were evaluated in model experiments using standard compounds, natural water and biomass as matrices.

### 5.4. Genotoxicity Testing

The Allium test was used to analyze genotoxic activity according to the standard procedure [30,31]. The onion bulbs (*Allium cepa* L., 2n = 16) were of the Stuttgarter variety, average weight 25 g. In the experiment, samples of water from microcosms of different treatments were used. Each treatment used 10 bulbs (80 for all groups in total). Bulbs of *A. cepa* were placed in small glass jars with their basal ends dipped in distilled water (control group) and in the water from the experimental treatments (experimental groups) and then germinated at room temperature (24 ± 3 °C) for 48 h. Then, roots were fixed in Clarke’s solution. For each group, 10 slides were prepared to analyze microscopic parameters. Aceto-orcein staining was used. For each treatment, at least 5000 cells were analyzed. Ana-telophase chromosomal aberration assays were applied to detect fragments and chromosome bridges in the total number of anaphases and telophases per whole slide. Micronuclei tests were applied to detect small and large micronuclei in the interphase cells. Micronuclei frequencies were expressed as the number of interphase cells with micronuclei per 3000 interphase cells for every slide. Mitotic abnormalities were registered such as lagging chromosomes, vagrant chromosomes and sticky chromosomes. Additionally, cells with c-mitosis were recorded. Mitotic abnormalities were scored in all anaphase and telophase cells per slide. Polyploidy frequencies were scored per 1000 dividing cells. The mitotic index was calculated for each slide as the number of dividing cells (prophases, metaphases, anaphases and telophases) per at least 500 cells, and the proportions of mitotic phases (prophase index, metaphase index, anaphase index and telophase index) were also assessed. Light microscopy at 400–1000× was used to analyze cells.

### 5.5. Data Analysis

The significance of differences in the frequency of occurrence of various types of cytogenetic effects in the experimental treatments and in the control was assessed using the Mann–Whitney criterion.

## Supplementary Materials

The following supporting information can be downloaded at: https://www.mdpi.com/article/10.3390/toxins14050359/s1, Table S1: Average concentration of MCs in cyanobacterial biomass (intracellular fraction), μg/L; Table S2: Average concentration of MCs in water, μg/L; Table S3: Genotoxicity data. Mann-Whitney test. (mean ± SE); Figure S1. Photography (made at 20 July 2021) of the model experiment (microcosms) with different compositions of their biotic components in plastic fish-breeding containers (on the basis of Stationary of Experimental Field and Expeditionary Works of the Papanin Institute for Biology of Inland Waters Russian Academy of Sciences); Figure S2. Photography (made at 20 July 2021) of pool with cyanobacterial blooming (on the basis of Stationary of Experimental Field and Expeditionary Works of the Papanin Institute for Biology of Inland Waters Russian Academy of Sciences).
