# Supplementary material for: Genotoxicity of Natural Water during the Mass Development of Cyanobacteria Evaluated by the Allium Test Method: A Model Experiment with Microcosms
Source: Toxins (Basel). 2022 May 21;14(5):359. doi: 10.3390/toxins14050359 (PMC9145725; doi:10.3390/toxins14050359)
Supplement: Supplementary file 1 [file toxins-14-00359-s001.zip › toxins-1724918-supplementary.pdf]

Supplementary Materials: Genotoxicity of Natural Water during the Mass Development of Cyanobacteria Evaluated by the Allium Test Method: A Model Experiment with Microcosms

Dmitry S. Pesnya, Svetlana A. Kurbatova, Andrey N. Sharov, Ekaterina N. Chernova, Igor Yu. Yershov, Galina V. Shurganova and Ekaterina L. Vodeneeva

Table S1. Average concentration of MCs in cyanobacterial biomass (intracellular fraction), μg/L.

Variant	Gross formula	[M+H]+	Treatment		
			ZP	Amph	F
July 19					
[D-Asp3]MC-LR	$C_{48}H_{72}N_{10}O_{12}$	981.54	0.338	0.353	0.409
MC-LR	$C_{49}H_{74}N_{10}O_{12}$	995.56	6.405	7.641	9.371
[D-Glu-OCH36]MC-LR	$C_{50}H_{76}N_{10}O_{12}$	1009.57	0.092	0.114	0.140
[D-Asp3]MC-RR	$C_{48}H_{73}N_{13}O_{12}$	1024.56	0.006	0.006	0.014
MC-LW	$C_{54}H_{72}N_{8}O_{12}$	1025.54	0.020	0.024	0.035
[Dha7]MC-YR	$C_{51}H_{70}N_{10}O_{13}$	1031.52	0.532	0.621	0.617
MC-RR	$C_{49}H_{75}N_{13}O_{12}$	1038.57	0.039	0.000	0.075
MC-YR	$C_{52}H_{72}N_{10}O_{13}$	1045.54	1.911	1.647	1.339
Total			9.343	10.405	12.001
August 19					
[D-Asp3]MC-LR	$C_{48}H_{72}N_{10}O_{12}$	981.54	0.222	0.056	0.454
MC-LR	$C_{49}H_{74}N_{10}O_{12}$	995.56	0.123	0.076	0.683
[D-Glu-OCH36]MC-LR	$C_{50}H_{76}N_{10}O_{12}$	1009.57	< 0.002	< 0.002	< 0.002
[D-Asp3]MC-RR	$C_{48}H_{73}N_{13}O_{12}$	1024.56	< 0.002	< 0.002	< 0.002
MC-LW	$C_{54}H_{72}N_{8}O_{12}$	1025.54	< 0.002	< 0.002	< 0.002
[Dha7]MC-YR	$C_{51}H_{70}N_{10}O_{13}$	1031.52	0.208	0.048	0.238

MC-RR	$C_{49}H_{75}N_{13}O_{12}$	1038.57	0.003	0.007	0.007
MC-YR	$C_{52}H_{72}N_{10}O_{13}$	1045.54	1.756	0.455	5.694
Total			2.311	0.641	7.076

Table S2. Average concentration of MCs in water, µg/L.

Variant	Gross formula	[M+H]+	Treatment		
			ZP	Amph	F
July 19					
[D-Asp³]MC-LR	$C_{48}H_{72}N_{10}O_{12}$	981.54	0.006	0.006	0.003
MC-LR	$C_{49}H_{74}N_{10}O_{12}$	995.56	0.007	0.091	0.082
[D-Glu-OCH₃⁶]MC-LR	$C_{50}H_{76}N_{10}O_{12}$	1009.57	0.054	0.071	0.064
[D-Asp³]MC-RR	$C_{48}H_{73}N_{13}O_{12}$	1024.56	< 0.002	< 0.002	< 0.002
MC-LW	$C_{54}H_{72}N_{8}O_{12}$	1025.54	< 0.002	< 0.002	< 0.002
[Dha⁷]MC-YR	$C_{51}H_{70}N_{10}O_{13}$	1031.52	0.006	0.005	0.003
MC-RR	$C_{49}H_{75}N_{13}O_{12}$	1038.57	< 0.002	< 0.002	< 0.002
MC-YR	$C_{52}H_{72}N_{10}O_{13}$	1045.54	0.006	0.054	0.032
Total			0.080	0.228	0.184
August 19					
[D-Asp³]MC-LR	$C_{48}H_{72}N_{10}O_{12}$	981.54	0.015	0.017	0.011
MC-LR	$C_{49}H_{74}N_{10}O_{12}$	995.56	0.063	0.085	0.043
[D-Glu-OCH₃⁶]MC-LR	$C_{50}H_{76}N_{10}O_{12}$	1009.57	0.027	0.026	0.047
[D-Asp³]MC-RR	$C_{48}H_{73}N_{13}O_{12}$	1024.56	< 0.002	< 0.002	< 0.002
MC-LW	$C_{54}H_{72}N_{8}O_{12}$	1025.54	< 0.002	< 0.002	< 0.002
[Dha⁷]MC-YR	$C_{51}H_{70}N_{10}O_{13}$	1031.52	0.012	0.012	0.005
MC-RR	$C_{49}H_{75}N_{13}O_{12}$	1038.57	< 0.002	< 0.002	< 0.002
MC-YR	$C_{52}H_{72}N_{10}O_{13}$	1045.54	0.079	0.114	0.123
Total			0.197	0.254	0.229

Table S3. Genotoxicity data. Mann-Whitney test. (mean±SE).

Treatment	Date	Mitotic Indexes	Phase Indexes				Polyploidy	Mitotic Abnormalities	Micronuclei	Chromosomal Aberrations
			prophase	metaphase	anaphase	telophase				
Control	July 19	6.52 ± 0.54<br>(5.52; 7.36)	45.18 ± 1.88<br>(41.87; 48.26)	19.08 ± 2.36<br>(14.22; 22.60)	19.89 ± 2.16<br>(16.00; 23.42)	15.85 ± 1.73<br>(13.02; 18.69)	0.02 ± 0.02<br>(0.00; 0.04)	0.30 ± 0.13<br>(0.08; 0.49)	0.04 ± 0.02<br>(0.00; 0.06)	0.22 ± 0.14<br>(0.00; 0.41)
ZP	July 19	5.66 ± 0.26<br>(5.29;6.13)	41.11 ± 2.92<br>(35.52;45.62)	**27.78 ± 2.21***<br>**(23.67;31.53)**	13.53 ± 1.00<br>(11.81;15.13)	17.59 ± 1.12<br>(15.45;19.39)	**0.30 ± 0.09***<br>**(0.14;0.44)**	**2.51 ± 0.61***<br>**(1.51;3.52)**	0.16 ± 0.05<br>(0.06;0.24)	0.31 ± 0.22<br>(0.00;0.62)
Amph	July 19	6.49 ± 1.29<br>(4.32;8.66)	39.53 ± 1.95<br>(36.25;42.67)	22.47 ± 1.60<br>(19.82;25.07)	18.24 ± 2.05<br>(15.19;22.04)	19.76 ± 2.22<br>(15.68;23.36)	**0.20 ± 0.03***<br>**(0.12;0.24)**	1.64 ± 0.86<br>(0.30;3.23)	0.08 ± 0.04<br>(0.00;0.12)	0.94 ± 0.30<br>(0.44;1.43)
F	July 19	7.88 ± 0.50<br>(6.97;8.74)	42.78 ± 1.79<br>(39.44;45.42)	28.17 ± 2.44<br>(23.47;31.65)	**11.21 ± 1.00***<br>**(9.42;12.90)**	17.84 ± 3.36<br>(13.40;24.02)	**0.54 ± 0.18***<br>**(0.22;0.82)**	**2.49 ± 0.78***<br>**(1.19;3.75)**	0.08 ± 0.05<br>(0.00;0.12)	0.93 ± 0.29<br>(0.34;1.40)
Control	Aug 19	7.52 ± 0.20<br>(7.22; 7.88)	41.31 ± 1.11<br>(39.15; 42.99)	25.36 ± 1.46<br>(22.61; 27.76)	15.87 ± 1.03<br>(13.97; 17.58)	17.46 ± 1.38<br>(15.24; 19.80)	0.06 ± 0.02<br>(0.00; 0.08)	0.20 ± 0.12<br>(0.00; 0.38)	0.06 ± 0.02<br>(0.00; 0.08)	0.24 ± 0.15<br>(0.00; 0.44)
ZP	Aug 19	6.71 ± 0.54<br>(5.70; 7.59)	40.11 ± 0.98<br>(38.43; 41.72)	27.35 ± 0.64<br>(26.21; 28.44)	17.16 ± 0.98<br>(15.32; 18.72)	15.38 ± 1.42<br>(12.93; 17.83)	0.16 ± 0.09<br>(0.00; 0.32)	0.29 ± 0.21<br>(0.00; 0.58)	0.08 ± 0.04<br>(0.00; 0.12)	0.83 ± 0.35<br>(0.23; 1.45)
Amph	Aug 19	7.35 ± 0.34<br>(6.70;7.91)	41.98 ± 0.78<br>(40.77;43.34)	25.93 ± 1.34<br>(23.29;28.16)	17.12 ± 0.63<br>(16.01; 18.23)	14.97 ± 0.68<br>(13.90; 16.11)	0.06 ± 0.02<br>(0.00;0.08)	0.09 ± 0.09<br>(0.00;0.19)	0.04 ± 0.02<br>(0.00;0.06)	0.50 ± 0.23<br>(0.10;0.87)
F	Aug 19	7.68 ± 0.38<br>(6.96; 8.23)	39.51 ± 1.60<br>(36.94; 42.24)	23.04 ± 1.15<br>(21.13;24.92)	16.49 ± 1.19<br>(14.34;18.75)	20.96 ± 1.25<br>(18.72;23.17)	**0.18 ± 0.04***<br>**(0.10; 0.24)**	**1.21 ± 0.50***<br>**(0.57; 2.16)**	0.06 ± 0.02<br>(0.00;0.08)	0.70 ± 0.37<br>(0.17;1.36)

Treatments: ZP – microcosms with only zooplankton, Amph – zooplankton and amphipods, F – zooplankton and fish. The differences that are significant in comparison with the control are highlighted in bold and with (*). (x; y) - 95% confidence interval.

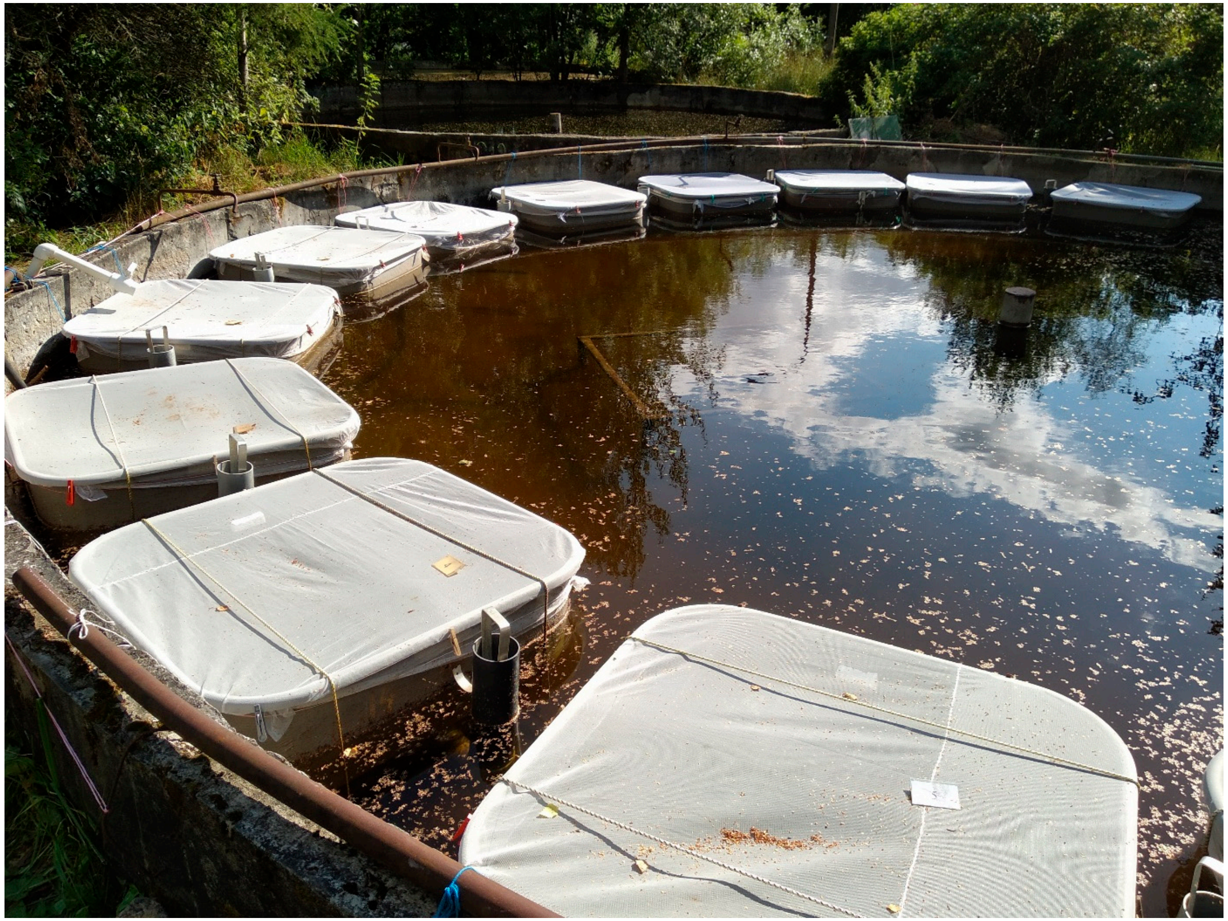

Figure S1. Photography (made at 20 July 2021) of the model experiment (microcosms) with different compositions of their biotic components in plastic fish-breeding containers (on the basis of Stationary of Experimental Field and Expeditionary Works of the Papanin Institute for Biology of Inland Waters Russian Academy of Sciences.).

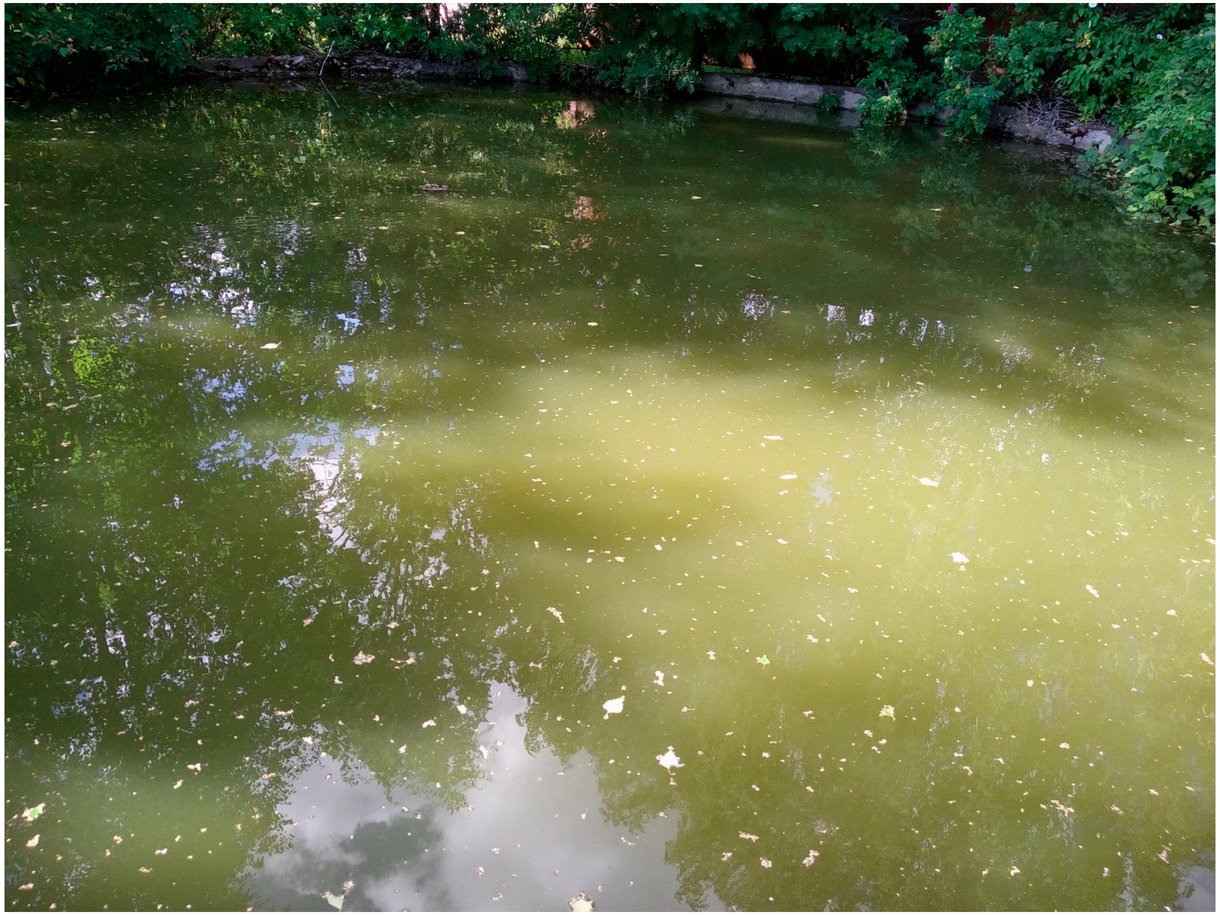

Figure S2. Photography (made at 20 July 2021) of pool with cyanobacterial blooming (on the basis of Stationary of Experimental Field and Expeditionary Works of the Papanin Institute for Biology of Inland Waters Russian Academy of Sciences.)
